# Pledgeted versus nonpledgeted sutures in aortic valve replacement: Insights from a prospective multicenter trial

**DOI:** 10.1016/j.xjtc.2022.10.016

**Published:** 2022-11-05

**Authors:** Bart J.J. Velders, Michiel D. Vriesendorp, Joseph F. Sabik, Francois Dagenais, Louis Labrousse, Vinayak Bapat, Gabriel S. Aldea, Anelechi C. Anyanwu, Yaping Cai, Robert J.M. Klautz

**Affiliations:** a Department of Cardiothoracic Surgery, Leiden University Medical Centre, Leiden, the Netherlands; b Department of Surgery, University Hospitals, Case Western Reserve University School of Medicine, Cleveland, Ohio; c Division of Cardiac Surgery, Quebec Heart and Lung Institute, Quebec, Quebec, Canada; d Medico-Surgical Department of Valvulopathies, Bordeaux Heart University Hospital, Bourdeaux-Pessac, France; e Department of Cardiothoracic Surgery, NHS Foundation Trust–St Thomas' Hospital, London, United Kingdom; f Department of Cardiothoracic Surgery, University of Washington School of Medicine, Seattle, Wash; g Department of Cardiovascular Surgery, Icahn School of Medicine at Mount Sinai, New York, NY; h Core Clinical Solutions, Medtronic, Mounds View, Minn

**Keywords:** pledgets, surgical aortic valve replacement, suturing technique, thromboembolism, endocarditis, paravalvular leak, AVR, aortic valve replacement, BMI, body mass index, BSA, body surface area, EOA, effective orifice area, EOAi, effective orifice area indexed, LVOT, left ventricular outflow tract, PERIGON, PERIcardial SurGical AOrtic Valve ReplacemeNt, PPM, prosthesis–patient mismatch, PVL, paravalvular leak, STS, Society of Thoracic Surgeons

## Abstract

**Objective:**

The objective of this study was to compare short- and midterm clinical and echocardiographic outcomes according to the use of pledgeted sutures during aortic valve replacement.

**Methods:**

Patients with aortic stenosis or regurgitation requiring aortic valve replacement were enrolled in a prospective cohort study to evaluate the safety of a new stented bioprosthesis. Outcomes were analyzed according to the use of pledgets (pledgeted group) or no pledgets (nonpledgeted group). The primary outcome was a composite of thromboembolism, endocarditis, and major paravalvular leak at 5 years of follow-up. Secondary outcomes included multiple clinical endpoints and hemodynamic outcomes. Propensity score matching was performed to adjust for prognostic factors, and subanalyses with small valve sizes (<23 mm) and suturing techniques were performed.

**Results:**

The pledgeted group comprised 640 patients (59%), and the nonpledgeted group 442 (41%), with baseline discrepancies in demographic characteristics, comorbidities, and stenosis severity. There were no differences between groups in any outcome. After propensity score matching, the primary outcome occurred in 41 (11.7%) patients in the pledgeted and 36 (9.8%) in the nonpledgeted group (*P* = .51). The effective orifice area was smaller in the pledgeted group (*P* = .045), whereas no difference was observed for the mean or peak pressure gradient. Separate subanalyses with small valve sizes and suturing techniques did not show relevant differences.

**Conclusions:**

In this large propensity score-matched cohort, comprehensive clinical outcomes were comparable between patients who underwent aortic valve replacement with pledgeted and nonpledgeted sutures up to 5 years of follow-up, but pledgets might lead to a slightly smaller effective orifice area in the long run.

## Graphical abstract

**Figure:**
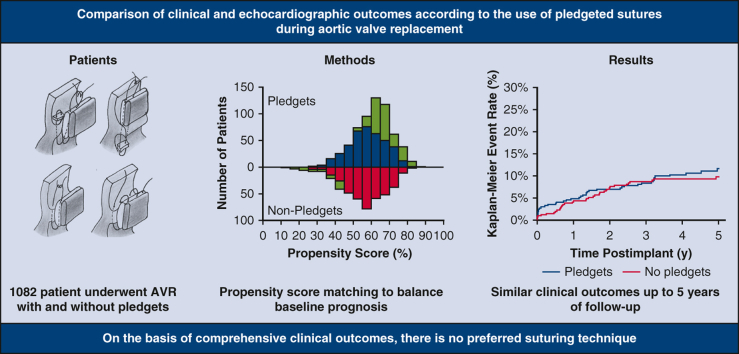

**Figure:**
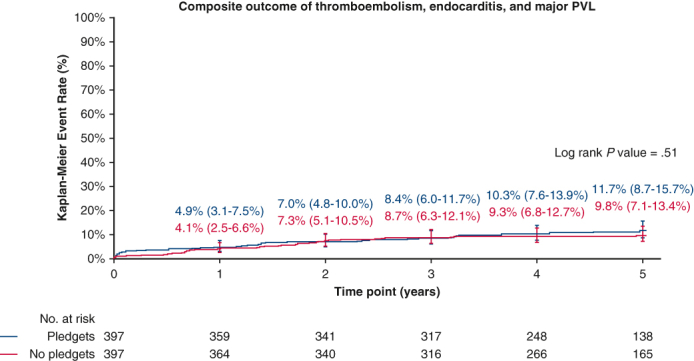
Five-year outcomes according to the use of pledgets in the propensity score-matched cohort.

**Central Message**

Clinical outcomes were comparable for patients who underwent aortic valve replacement (AVR) with and without pledgets.

**Perspective**

Whether to use pledgets for surgical AVR is an ongoing debate among surgeons. In a propensity score-matched analysis, comprehensive clinical outcomes were comparable between patients who underwent AVR with pledgeted and nonpledgeted sutures up to 5 years of follow-up. Nevertheless, pledgets might lead to a slight reduction of the EOA in the long run, but this finding requires external validation.

Aortic valve replacement (AVR) is the second-most commonly performed type of cardiac surgery, and rates are increasing because of an aging population.^1^ Although AVR has been performed and improved over several decades, there is still debate among surgeons about the optimal implantation technique. An interesting topic that lacks consensus is whether to use pledgeted sutures to secure the prosthetic valve, because the literature shows conflicting results (Table 1).

**Table 1:** Overview of previous studies regarding the use of pledgets in aortic valve replacement

Study characteristics					Hemodynamic performance			Clinical outcomes		
Reference	Design	Valve	N	FU length, mo	MPG, mm Hg	EOA, cm^2^	PVL	Operative mortality	TE	IE
Englberger et al.^2^	RCT secondary analysis	Mechanical (aortic/mitral)	807	60	–	–	1.7% PS vs 5.8% NPS. HR, 0.3 for PS (*P* < .01)	–	–	–
LaPar et al.^3^	Retrospective cohort	Biological, mechanical, homograft	802	82	–	–	PS 1.2% vs NPS 0.5% (*P* = .38)	PS 2.3% vs NPS 1.9% (*P* = .79)	–	–
Tabata et al.^4^	Retrospective cohort	Biological (19-21 mm)	152	12	–	Postimplantation: PS 1.30 ± 0.28 vs NPS 1.42 ± 0.32 (*P* = .03). 1 y: No difference (*P* = .13)	No difference (*P* > .99)	–	–	–
Ugur et al.^5^	Prospective cohort	Biological (19-21 mm)	346	12	PS 8.9 ± 3.9 vs NPS 9.6 ± 4.1 (*P* = .16)	1 y: PS 1.53 ± 0.3 vs NPS 1.42 ± 0.3 (*P* = .04)	No difference (*P* = NA)	–	–	–
Kim et al.^6^	Retrospective cohort	Biological, mechanical	439	12	–	1 y: PS 1.74 ± 1.38 vs NPS 1.70 ± 0.34 vs figure-of-eight 1.7 ± 0.42 (*P* = .97)	PS 0.5% vs NPS 0% vs figure-of-eight 1% (*P* = .99)	PS 2.4% vs NPS 2.5% vs figure-of-eight 5.7% (*P* = .28)	PS 0.5% vs NPS 0.8% vs figure-of-eight 0% (*P* = .44)	–

*FU*, Follow-up; *MPG*, mean pressure gradient; *EOA*, effective orifice area; *PVL*, paravalvular leak; *TE*, thromboembolism; *IE*, infective endocarditis; *RCT*, randomized controlled trial; *PS*, pledgeted sutures; *NPS*, nonpledgeted sutures; *HR*, hazard ratio; *NA*, not available.

Some argue that the use of pledgeted sutures allow for more even distribution of mechanical forces and a tighter connection between the prosthesis and the aortic annulus/root, thereby decreasing the incidence of paravalvular leak (PVL).^2^ However, others believe that pledgets create an additional level of obstruction in the left ventricular outflow tract (LVOT), leading to a higher transvalvular gradient, a smaller effective orifice area (EOA),^4^^,^^5^ and subsequently more frequent prosthesis–patient mismatch (PPM).^6^ Theoretically, the use of pledgets could also induce higher rates of thromboembolism or endocarditis due to extra foreign material.

Within the PERIcardial SurGical AOrtic Valve ReplacemeNt (PERIGON) Pivotal Trial of the Avalus bioprosthesis (Medtronic), the technical details for implantation were left to the discretion of the surgeon. We aimed to provide insight into the effect of pledgeted sutures during AVR on multiple clinical and hemodynamic outcomes. The primary outcome of interest was a composite of thromboembolism, endocarditis, and major PVL at 5-year follow-up.

## Methods

### Study Design

The PERIGON Pivotal Trial (www.clinicaltrials.gov, NCT02088554) is a prospective multicenter trial that is conducted at 38 sites across the United States, Canada, and Europe. In this single-armed trial, clinical and hemodynamic outcomes of the Avalus bioprosthesis (Medtronic), a stented bovine pericardial aortic valve, are evaluated. The study design was previously described in detail.^7^^,^^8^ In short, symptomatic patients with moderate or severe aortic stenosis or chronic, severe aortic regurgitation who were admitted for surgical AVR according to clinical indication were enrolled. Patients with and without concomitant procedures, limited to coronary artery bypass grafting, left atrial appendage ligation, patent foramen ovale closure, ascending aortic aneurysm or dissection repair not requiring circulatory arrest, and subaortic membrane resection not requiring myectomy, were included. In the PERIGON Pivotal Trial protocol, surgical technical details were left to the surgeon's own consideration.

The trial was conducted according to the Declaration of Helsinki and good clinical practice. At each site, approval of the protocol was obtained from the institutional review board or ethics committee (Table E1), and written informed consent was provided by all patients. All deaths and valve-related adverse events were adjudicated by an independent clinical events committee, and study oversight was provided by an independent data and safety monitoring board (Baim Institute for Clinical Research). All echocardiographic data were evaluated by an independent core laboratory (MedStar).

In the present study, patients were stratified to noneverted or everted mattress sutures with pledgets (pledgeted group), and noneverted or everted mattress, continuous, or simple interrupted sutures without pledgets (nonpledgeted group). Patients with previous aortic valve implantation (n = 10), figure-of-eight sutures (n = 3), or noncategorized sutures (n = 23) were excluded.

### Follow-up and End Points

Annual clinical and (transthoracic) echocardiographic evaluations were performed after the first year of follow-up. Patient and procedural characteristics, early outcomes (within 30 days postimplantation), and 5-year outcomes were compared among the pledgeted and nonpledgeted groups. The primary outcome was a composite of thromboembolism, endocarditis, and major PVL at 5-year follow-up. Other clinical parameters included in the early- and midterm outcome analysis consisted of mortality, thromboembolism, endocarditis, all and major hemorrhage, all and major PVL, explant, reintervention, and permanent pacemaker implantation.

Echocardiographic outcomes consisted of mean and peak pressure gradients calculated using the simplified Bernoulli formula, and EOA, which was determined using the continuity equation. EOA indexed (EOAi) by body surface area (BSA) was used to classify PPM. PPM was defined according to the Valve Academic Research Consortium 3 criteria as insignificant (EOAi >0.85 cm^2^/m^2^ or >0.70 cm^2^/m^2^), moderate (EOAi between 0.85 and 0.66 cm^2^/m^2^ or 0.70 and 0.56 cm^2^/m^2^), or severe (EOAi ≤0.65 cm^2^/m^2^ or ≤0.55 cm^2^/m^2^) for patients with a body mass index (BMI) <30 or ≥30, respectively.^9^

### Statistical Analysis

Continuous variables are presented as mean ± SD and categorical variables as number and percentage. The independent sample *t* test or Mann–Whitney *U* test was used to compare continuous variables, and χ^2^ or Fisher exact test was used for categorical variables. Early and 5-year clinical event rates (including 95% CI) were summarized using the Kaplan–Meier method, and the log rank test was used to calculate *P* values. An additional evaluation of hemodynamic performance postimplantation and at 5-year follow-up in valve sizes smaller than 23 mm was performed. Furthermore, hemodynamic performance according to suturing techniques within the nonpledgeted group were compared for the “mattress” (noneverted and everted mattress sutures) and “nonmattress” (continuous and simple interrupted sutures) groups to investigate differences not related to the use of pledgets.

Propensity score matching was performed to account for potential bias arising from the decision to use pledgets. Propensity scores were calculated on the basis of the following variables: age, male sex, BSA, Society of Thoracic Surgeons (STS) risk of mortality, New York Heart Association class III/IV, coronary artery disease, chronic obstructive pulmonary disease, hypertension, previous myocardial infarction, renal dysfunction/insufficiency, diabetes mellitus, atrial fibrillation, peripheral vascular disease, previous stroke/cerebrovascular accident, left ventricular ejection fraction at baseline, mean pressure gradient at baseline, isolated/mixed aortic stenosis, and less invasive approach (hemisternotomy or right anterior thoracotomy). Baseline left ventricular ejection fraction and baseline mean pressure gradient were missing for 225 (20.8%) and 26 (2.4%) patients, respectively. To avoid losing patients in the postmatched analysis, the missing values were imputed with the median before entering propensity score matching. A 5-to-1 digits greedy 1:1 matching algorithm was used to form a propensity score-matched cohort for analysis.

A 2-sided α level of 0.05 was used in all tests. The balance in baseline characteristics before and after propensity score matching was expressed in standardized mean differences. Statistical analyses were performed with SAS version 9.4 (SAS Institute Inc).

## Results

### Entire Cohort

Six hundred forty (59%) patients underwent AVR with pledgeted sutures, and 442 (41%) underwent AVR with nonpledgeted sutures. The baseline characteristics are summarized in Table 2. Baseline differences existed in age, BSA, BMI, STS risk of mortality, hypertension, left ventricular hypertrophy, atrial fibrillation, isolated or mixed aortic stenosis as the primary indication for AVR, minimally invasive surgical approach, concomitant procedures, and implanted valve sizes. At 30 days, all clinical and hemodynamic end points were comparable (Table E2). At 5 years of follow-up, the composite outcome of thromboembolism, endocarditis, and major PVL occurred in 9.2% of the pledgeted group and 10.2% of the nonpledgeted group (*P* = .59; Table E3). Moreover, there were no differences in the separate components of the composite outcome, nor in other clinical or hemodynamic outcomes.

**Table 2:** Baseline and procedural characteristics according to the use of pledgets for patients who underwent aortic valve replacement in the entire cohort and the propensity score-matched cohort

	Entire cohort (N = 1082)			Propensity score-matched cohort (n = 794)		
	Pledgets (n = 640)	No pledgets (n = 442)	SMD	Pledgets (n = 397)	No pledgets (n = 397)	SMD
Age, y	69.6 ± 8.5	71.0 ± 9.4	0.148	70.2 ± 8.3	70.3 ± 9.2	0.010
Male sex	494 (77.2)	323 (73.1)	0.095	300 (75.6)	295 (74.3)	0.029
Body surface area, m^2^	2.01 ± 0.2	1.96 ± 0.2	0.205	1.98 ± 0.2	1.98 ± 0.2	0.019
Body mass index	29.8 ± 5.5	29.0 ± 5.3	0.145	29.4 ± 5.7	29.2 ± 5.4	0.026
NYHA classification III-IV	272 (42.5)	189 (42.8)	0.005	158 (39.8)	166 (41.8)	0.041
STS risk of mortality, %	1.9 ± 1.2	2.1 ± 1.6	0.211	1.90 ± 1.20	1.90 ± 1.24	0.004
Diabetes	179 (28.0)	114 (25.8)	0.049	108 (27.2)	99 (24.9)	0.052
Hypertension	510 (79.7)	318 (71.9)	0.182	293 (73.8)	291 (73.3)	0.011
Peripheral vascular disease	40 (6.3)	39 (8.8)	0.098	26 (6.5)	31 (7.8)	0.049
Renal dysfunction/insufficiency	65 (10.2)	50 (11.3)	0.037	48 (12.1)	40 (10.1)	0.064
Stroke/CVA	28 (4.4)	16 (3.6)	0.039	10 (2.5)	13 (3.3)	0.045
COPD	79 (12.3)	48 (10.9)	0.046	45 (11.3)	42 (10.6)	0.024
Left ventricular ejection fraction, %	59.8 ± 9.0	58.6 ± 10.1	0.126	58.67 ± 9.5	59.71 ± 9.0	0.112
Coronary artery disease	288 (45.0)	183 (41.4)	0.073	167 (42.1)	168 (42.3)	0.005
Left ventricular hypertrophy	284 (44.4)	161 (36.4)	0.163	160 (40.3)	146 (36.8)	0.073
Atrial fibrillation	52 (8.1)	59 (13.3)	0.169	45 (11.3)	41 (10.3)	0.032
Isolated/mixed aortic stenosis	597 (93.3)	425 (96.2)	0.129	380 (95.7)	382 (96.2)	0.026
Minimally invasive surgical approach	150 (24.3)	70 (16.5)	0.200	76 (19.1)	70 (17.6)	0.010
Concomitant procedure						
None	288 (45.0)	242 (54.8)	0.196	175 (44.1)	218 (54.9)	0.218
CABG	223 (34.8)	128 (29.0)	0.127	145 (36.5)	115 (29.0)	0.162
Ascending aortic aneurysm not requiring circulatory arrest	48 (7.5)	35 (7.9)	0.016	30 (7.6)	32 (8.1)	0.019
Other∗	161 (25.2)	68 (15.4)	0.245	92 (23.2)	58 (14.6)	0.220
Annular calcification	516 (80.6)	371 (83.9)	0.16	320 (80.6)	331 (83.4)	0.072
Total bypass time, min	104.2 ± 40.6	105.6 ± 41.0	0.035	101.7 ± 38.4	105.8 ± 41.2	0.103
Aortic crossclamp time, min	79.2 ± 31.2	79.5 ± 32.3	0.012	78.2 ± 30.0	79.9 ± 32.4	0.052
Annular diameter†	23.7 ± 2.05	23.7 ± 2.17	0.021	23.7 ± 2.13	23.7 ± 2.19	0.019
Valve size implanted						
17 mm	0 (0.0)	1 (0.2)	0.067	0 (0.0)	0 (0.0)	0.000
19 mm	16 (2.5)	23 (5.2)	0.141	8 (2.0)	20 (5.0)	0.164
21 mm	115 (18.0)	88 (19.9)	0.050	79 (19.9)	75 (18.9)	0.025
23 mm	226 (35.3)	161 (36.4)	0.023	145 (36.5)	147 (37.0)	0.010
25 mm	216 (33.8)	126 (28.5)	0.113	125 (31.5)	114 (28.7)	0.060
27 mm	62 (9.7)	36 (8.1)	0.054	38 (9.6)	34 (8.6)	0.035
29 mm	5 (0.8)	7 (1.6)	0.074	2 (0.5)	7 (1.8)	0.119
Mean pressure gradient, mm Hg	41.7 ± 17.0	43.3 ± 16.8	0.096	43.3 ± 16.9	43.3 ± 16.7	0.001
Effective orifice area, cm^2^	0.78 (0.36-4.67)	0.75 (0.35-3.43)	0.164	0.75 (0.36-3.44)	0.76 (0.35-3.43)	0.013
Indexed effective orifice area, cm^2^/m^2^	0.39 (0.17-2.52)	0.38 (0.18-1.82)	0.131	0.38 (0.17-1.83)	0.39 (0.18-1.82)	0.013

Data are presented as mean ± SD, median (interquartile range), or n (%) except where otherwise noted. *SMD*, Standardized mean difference; *NYHA*, New York Heart Association; *STS*, Society of Thoracic Surgeons; *CVA*, cerebrovascular accident; *COPD*, chronic obstructive pulmonary disease; *CABG*, coronary artery bypass grafting.

∗ Includes implantable cardiac device, left atrial appendage closure, patent foramen ovale closure, resection of subaortic membrane not requiring myectomy, and dissection repair not requiring circulatory arrest.

† The annual diameter was determined intraoperatively and corresponds to the size of the replica end of the valve sizer.

**Figure E1:**
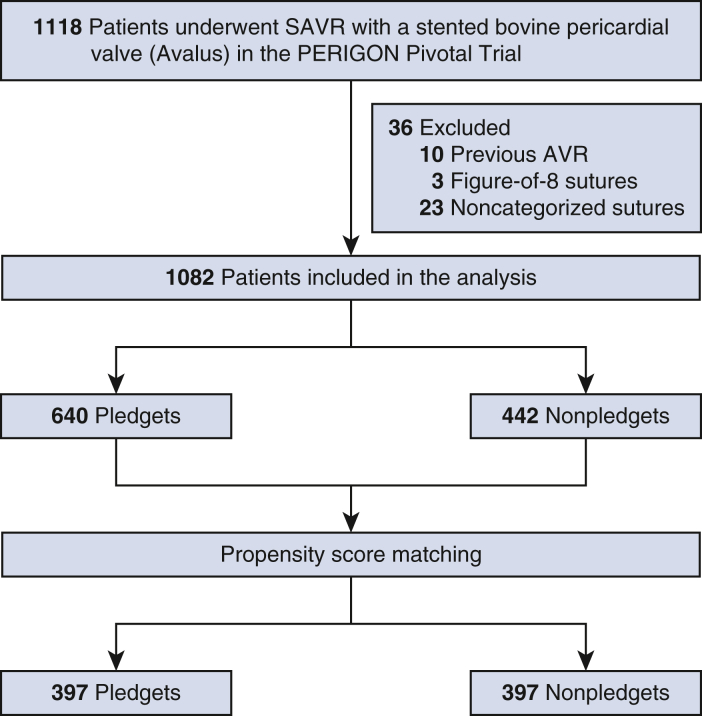
Consolidated Standards of Reporting Trials diagram of patients who underwent surgical aortic valve replacement with or without pledgeted sutures. The Avalus bioprosthesis is from Medtronic. *SAVR*, Surgical aortic valve replacement; *PERIGON*, PERIcardial SurGical AOrtic Valve ReplacemeNt; *AVR*, aortic valve replacement.

After propensity score matching, 794 patients (397 matched pairs) were eligible for the analysis (Figure E1). The groups were similar with regard to comorbidities and hemodynamic parameters, yet differences in concomitant procedures persisted (Table 2). At 30 days, the composite outcome was 2.8% in the pledgeted group and 1.0% in the nonpledgeted group (*P* = .07; Table E4). The hemodynamic parameters were similar between the 2 groups.

**Figure 1:**
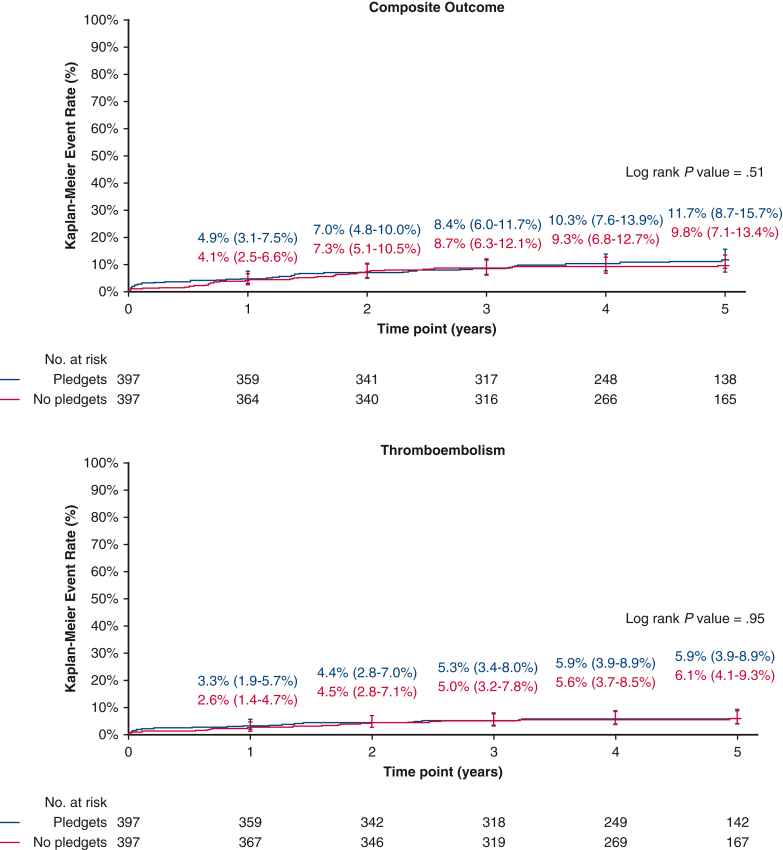
Kaplan–Meier event rates according to the use of pledgets for patients who underwent aortic valve replacement in the propensity score-matched cohort. Displayed are event rates for the composite outcome of thromboembolism, endocarditis, and major paravalvular leak (*top*), and for thromboembolism (*bottom*). The *whiskers* represent the 95% CI.

**Figure 2:**
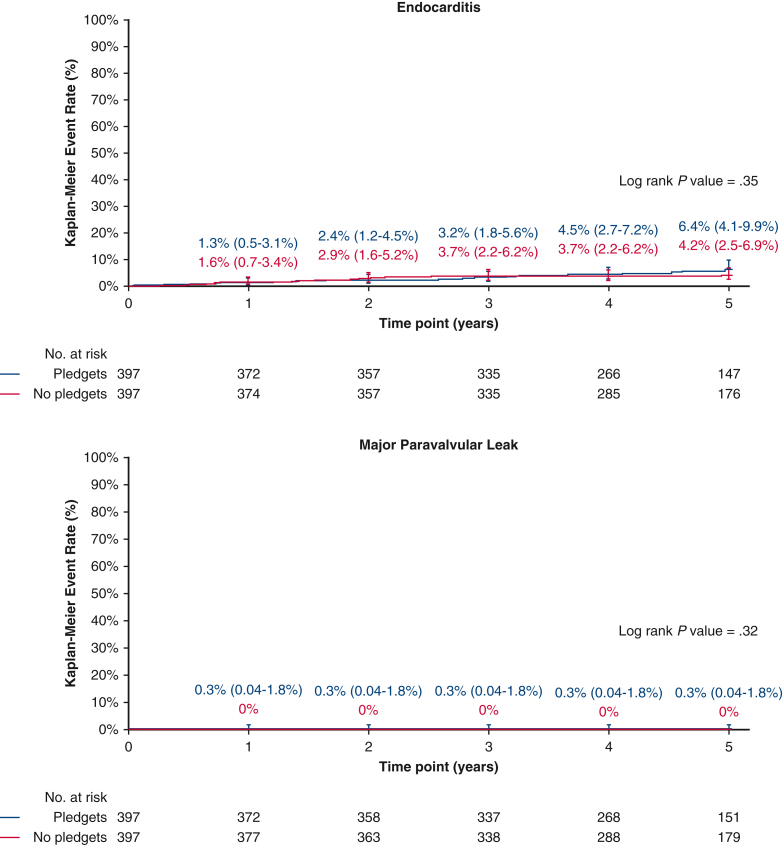
Kaplan–Meier event rates according to the use of pledgets for patients who underwent aortic valve replacement in the propensity score-matched cohort. Displayed are event rates for endocarditis (*top*), and for major paravalvular leak (*bottom*). The *whiskers* represent the 95% CI.

**Figure 3:**
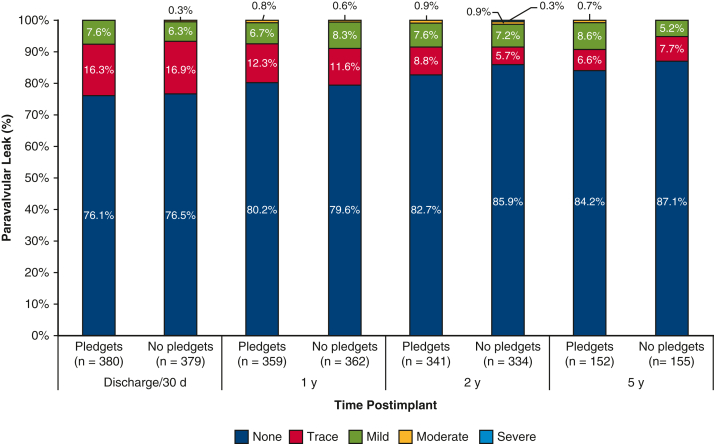
Paravalvular leak over time according to the use of pledgets for patients who underwent aortic valve replacement in the propensity score-matched cohort. The frequencies of paravalvular leak severity categories at different time points are displayed as *stacked bars*.

**Figure E2:**
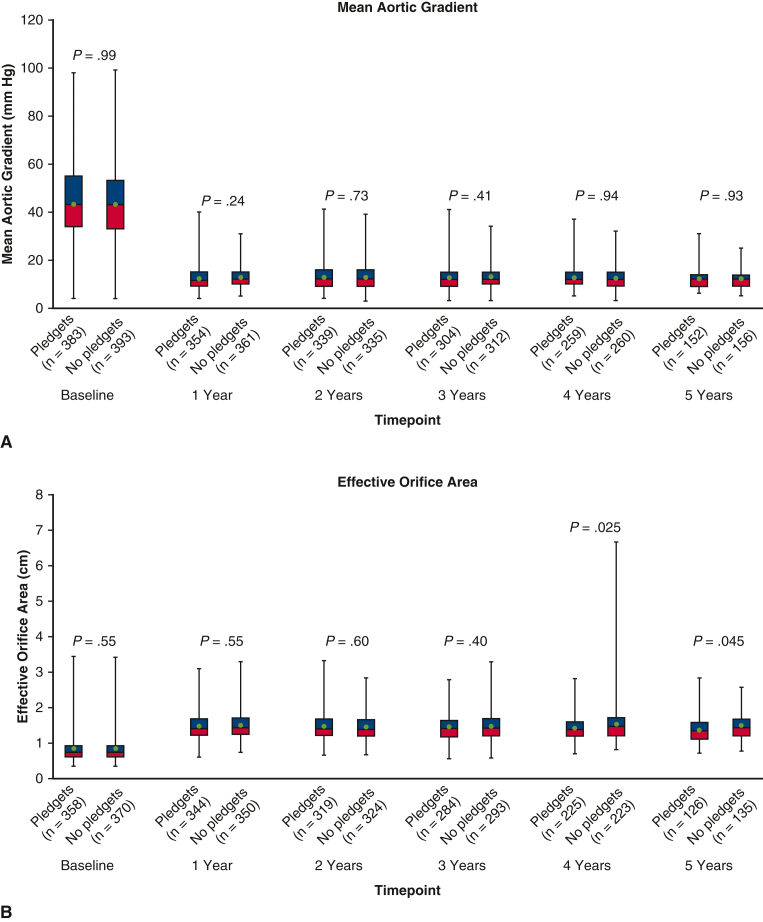
Hemodynamic performance over time according to the use of pledgets for patients who underwent aortic valve replacement in the propensity score-matched cohort. The *box plots* depict the (A) mean aortic gradient and (B) effective orifice area over time. Data are core lab reported. The *boxes* are centered at the median, with *upper and lower bounds of the box* being the 75th and 25th percentiles, respectively. The *upper and lower ends of the whiskers* represent maximum and minimum values. The *circle* represents the mean.

At 5 years of follow-up (Table 3), the composite outcome of thromboembolism, endocarditis, and major PVL occurred in 11.7% of the pledgeted group and in 9.8% of the nonpledgeted group (*P* = .51). The separate components were also comparable (Figures 1 and 2). The EOA was smaller in the pledgeted group (*P* = .045), but no difference was observed for the mean or peak pressure gradient. The mean pressure gradient remained stable over time, whereas the EOA decreased especially in the pledgeted group (Figure E2). The degree of PVL was consistent throughout follow-up (Figure 3). The proportion of patients with any PPM at 5-year follow-up was similar between the groups (Table 3).

**Table 3:** Clinical outcomes and hemodynamic performance at 5 years of follow-up for patients who underwent aortic valve replacement in the propensity score-matched cohort

	Pledgets (n = 397)	No pledgets (n = 397)	*P* value∗
Composite endpoint (thromboembolism, endocarditis, and major PVL)	11.7% (8.7%-15.7%) (n = 41)	9.8% (7.1%-13.4%) (n = 36)	.51
Thromboembolism	5.9% (3.9%-8.9%) (n = 22)	6.1% (4.1%-9.3%) (n = 22)	.95
Endocarditis	6.4% (4.1%-9.9%) (n = 20)	4.2% (2.5%-6.9%) (n = 15)	.35
Major PVL	0.3% (0.0%-1.8%) (n = 1)	0.0% (NA) (n = 0)	.32
All PVL	1.1% (0.4%-2.8%) (n = 4)	1.5% (0.5%-4.0%) (n = 4)	.96
All-cause mortality	13.3% (10.0%-17.6%) (n = 45)	10.5% (7.7%-14.2%) (n = 37)	.30
Cardiac-related mortality	6.8% (4.4%-10.3%) (n = 22)	4.2% (2.5%-7.1%) (n = 14)	.15
Valve-related mortality	2.2% (1.1%-4.4%) (n = 8)	0.5% (0.1%-2.1%) (n = 2)	.06
Reintervention	3.1% (1.7%-5.5%) (n = 11)	3.9% (2.2%-6.7%) (n = 13)	.74
Explant	3.1% (1.7%-5.5%) (n = 11)	3.2% (1.7%-5.7%) (n = 11)	.95
Permanent pacemaker implantation	5.6% (3.7%-8.5%) (n = 21)	6.9% (4.6%-10.1%) (n = 25)	.55
Mean pressure gradient, mm Hg	12.3 ± 4.4	12.3 ± 4.0	.93
Peak pressure gradient, mm Hg	22.0 ± 7.4	21.9 ± 7.4	.93
EOA, cm^2^	1.35 (0.72-2.87)	1.44 (0.79-2.58)	.045
EOAi, cm^2^/m^2^	0.69 (0.38-1.31)	0.73 (0.41-1.31)	.06
Prosthesis-patient mismatch			.07
None	40 (31.7%)	44 (32.6%)	
Moderate	46 (36.5%)	64 (47.4%)	
Severe	40 (31.7%)	27 (2.0%)	

Clinical outcomes are reported as 5-year Kaplan–Meier event rates, including 95% CI. Hemodynamic performance is presented either as mean ± SD or median (interquartile range). *PVL*, Paravalvular leak; *NA*, not available; *EOA*, effective orifice area; *EOAi*, effective orifice area indexed according to body surface area.

∗ *P* value from log rank test for all clinical outcomes and from independent samples *t* test, Mann–Whitney *U* test, or χ^2^ test for echocardiographic data.

### Subanalysis: Valve Sizes <23 mm

The baseline and procedural characteristics of patients with implanted valve sizes <23 mm are presented in Table E5. Pledgets were used in 131 patients, and no pledgets in 112 patients. As observed in the entire cohort, differences among the groups existed in baseline age, STS risk of mortality, concomitant procedures, and implanted valve size. Additionally, the aortic crossclamp time was longer in the pledgeted group than in the nonpledgeted group (78.6 ± 29.4 vs 69.2 ± 31.3 minutes; *P* = .017). The hemodynamic performance up to 30 days and at 5-year follow-up is shown in Table 4. The mean pressure gradient up to 30 days was lower in the pledgeted group compared with the nonpledgeted group (14.9 ± 4.6 vs 16.4 ± 5.6; *P* = .027), but this difference was absent at 5-year follow-up. All other parameters were comparable at both follow-up points.

**Table 4:** Hemodynamic performance at discharge up to 30 days and at 5 years of follow-up in valve sizes <23 mm for patients who underwent aortic valve replacement

	Pledgets (n = 131)	No pledgets (n = 112)	*P* value
Mean pressure gradient, mm Hg			
Discharge up to 30 days	14.9 ± 4.6	16.4 ± 5.6	.027
5 years	15.7 ± 5.6	15.0 ± 4.2	.50
Peak pressure gradient, mm Hg			
Discharge up to 30 days	27.5 ± 8.7	29.8 ± 9.8	.07
5 years	27.6 ± 9.2	26.1 ± 8.0	.38
Effective orifice area, cm^2^			
Discharge up to 30 days	1.31 (0.78-2.54)	1.29 (0.70-2.24)	.43
5 years	1.09 (0.72-1.95)	1.10 (0.79-1.70)	.54
Indexed effective orifice area, cm^2^/m^2^			
Discharge up to 30 days	0.72 (0.40-1.33)	0.70 (0.31-1.24)	.81
5 years	0.61 (0.43-1.05)	0.64 (0.43-1.04)	.47
Prosthesis-patient mismatch			
Discharge up to 30 days			.79
None	42 (35.9)	28 (31.5)	
Moderate	43 (36.8)	36 (4.4)	
Severe	32 (27.4)	25 (28.1)	
5 years			.50
None	3 (7.3)	6 (12.8)	
Moderate	16 (39.0)	21 (44.7)	
Severe	22 (53.7)	20 (42.6)	
Paravalvular leak			
Discharge up to 30 days			.60
None	76 (59.8)	70 (66.0)	
Trace	37 (29.1)	27 (25.5)	
Mild	14 (11.0)	9 (8.5)	
Moderate	0 (0.0)	0 (.0)	
Severe	0 (0.0)	0 (.0)	
5 years			.33
None	41 (83.7)	38 (79.2)	
Trace	3 (6.1)	7 (14.6)	
Mild	5 (10.2)	3 (6.3)	
Moderate	0 (0.0)	0 (0.0)	
Severe	0 (0.0)	0 (0.0)	

Numerical data are presented as mean ± SD or median (interquartile range) according to their distribution, and categorical data are summarized as n (%). Data were compared using the independent samples *t* test, Mann–Whitney *U* test, and χ^2^ test/Fisher exact test, respectively.

### Subanalysis: Nonpledgeted Sutures

Stratification of patients within the nonpledgeted group resulted in 180 patients in the mattress subgroup and 205 in the nonmattress subgroup. Their baseline characteristics are summarized in Table E6. Differences were observed in BMI, New York Heart Association class III/IV, diabetes mellitus, hypertension, renal dysfunction/insufficiency, stroke/cerebrovascular accident, chronic obstructive pulmonary disease, coronary artery disease, left ventricular hypertrophy, and concomitant procedures. The hemodynamic performance up to 30 days and at 5-year follow-up is presented in Table E7. At both time points, no differences related to suturing technique were found in echocardiographic variables, PPM, or PVL.

## Discussion

In a propensity score-matched analysis of a large international cohort, clinical outcomes at 30 days and 5 years of follow-up were comparable among patients who underwent surgical AVR with and without pledgeted sutures. Comparisons of pledgeted with nonpledgeted sutures in AVR in previous literature have mainly focused on hemodynamic performance (Table 1). Hence, insight into clinical outcomes is scarce. A potential disadvantage of pledgeted sutures is an increased risk of infection, pannus, or thrombus formation due to the presence of extra foreign material. A single study^6^ evaluated thromboembolism rates, whereas endocarditis has never been studied to our knowledge. In our analysis, both adverse events rarely occurred within 30 days of follow-up and were comparable at 5 years. Thus, there was no evidence of higher rates of these events when pledgets were used.

PVL is another important variable in the choice whether to use pledgeted sutures. Several studies have investigated this parameter but have reported conflicting results. Englberger and colleagues^2^ reported a reduction in PVL in the pledgeted sutures group. On the contrary, others reported no differences compared with nonpledgeted or figure-of-eight sutures.^4^, ^5^, ^3^, ^6^ Our findings were in line with the latter studies.

Regarding other hemodynamic performance measures such as the EOA, previous results were ambiguous, too. Tabata and colleagues^4^ observed a smaller EOA postimplantation in the pledgeted group that disappeared at 1 year, whereas Ugur and colleagues^5^ described a larger EOA at that time point. In the current study, the EOA was equal between the groups at short-term follow-up; however, at 5 years a difference appeared as a result of a smaller EOA in the pledgeted group. This phenomenon might be due to subvalvular obstruction caused by the pledgets and tissue (pannus) formation/ingrowth developing over time, which could lead to elevated velocities in the LVOT. Theoretically, such obstruction would be more profound in a small LVOT because pledgets have a fixed size, but in our subanalysis of valve sizes <23 mm, the EOAs were similar between the pledgeted and nonpledgeted groups (Table 4). Another explanation could be related to measurement error because the smaller EOA was not reflected by the mean or peak pressure gradient. Measurement of the LVOT diameter is prone to error and has a drastic effect on the EOA value because this diameter is squared to obtain the LVOT area for the continuity equation. The presence of pledgets might complicate the echocardiographic measurement of the LVOT diameter even more when it is examined in close proximity to the aortic annulus. Because the absolute difference in EOA was <0.1 cm^2^, the difference was absent in small valve sizes, and other hemodynamic parameters were equal between the groups, the clinical relevance of this difference in EOA is questionable. External validation of this finding and longer follow-up could provide valuable insights. A derivative of the indexed EOA is PPM. Because previous PERIGON substudies challenged the clinical relevance of this concept by outlining shortcomings regarding correspondence with elevated gradient and disproportional normalization by BSA,^10^, ^11^, ^12^ we chose to mainly elaborate on primary echocardiographic parameters rather than PPM in this study.

Although similar pressure gradients at 5 years were observed, a difference with lower values in the pledgeted group was found at 30 days, however, this dissimilarity was <1 mm Hg. Hence, it was not considered clinically important. To further investigate differences related to suturing technique, a subanalysis was executed within the nonpledgeted group. This analysis did not show any difference in the mattress and nonmattress suturing techniques.

Hemodynamic outcomes have received specific attention in smaller valve sizes. Two earlier studies reported similar hemodynamic parameters for pledgeted and nonpledgeted sutures.^4^^,^^5^ Our results are in agreement with these findings.

### Strengths and Limitations

A major advantage of the current study was that all 1082 patients received the same bioprosthetic valve, which eliminated any bias due to the type of prosthesis. Furthermore, the prospective design with independent adverse event adjudication and core laboratory assessment of echocardiograms enabled robust and consistent data-gathering up to 5 years of follow-up. Despite these strengths, there were limitations. Even though there was apparent harmony in patient characteristics after propensity score-matching, the study design could not guarantee complete comparability because adjustment was possible only for measured confounders. Specifically, we did not adjust for surgeon bias, and it is possible that surgeons who opted for one technique versus another might have different skills, leading to an inextricable confounding effect. The 1082 AVR procedures in this analysis were performed by 132 surgeons, some of whom solely used pledgeted (54 surgeons) or nonpledgeted sutures (33 surgeons). Hence, we did not incorporate surgeon data in the propensity score matching. To achieve complete comparability, randomized treatment allocation would have been a prerequisite, which was not the case. Furthermore, no correction methods were applied to the subanalyses, in which the statistical power was also decreased because of smaller sample sizes. Therefore, these results should be interpreted in the context of these limitations. An increased length of follow-up might have revealed more profound differences in outcomes. It would be of interest to observe whether the difference in EOA will persist and eventually lead to differences in clinical outcomes such as reintervention. Important aspects that remain unknown to the discussion of whether to use pledgeted sutures for surgical AVR are the feasibility of reoperations and future valve-in-valve transcatheter AVR for degenerated bioprostheses. Unfortunately, no quantitative claims can be made on the basis of data from the current study. For future studies on this topic, these issues are highly relevant.

## Conclusions

**Figure 4:**
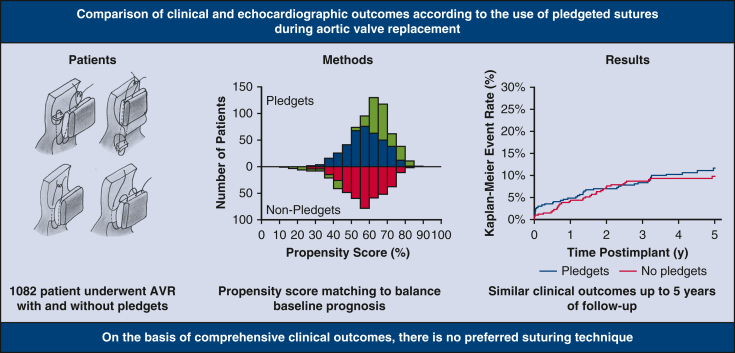
Pledgeted versus nonpledgeted sutures in aortic valve replacement: insights from a prospective multicenter trial. Outcomes were compared according to the use of pledgeted sutures. Propensity score matching was used to adjust for baseline differences. The images showing the suturing techniques were reproduced from Kirali and colleagues,^13^ with permission from Elsevier. *AVR*, Aortic valve replacement.

In a propensity score-matched analysis, comprehensive clinical outcomes were comparable between patients who underwent AVR with pledgeted and nonpledgeted sutures up to 5 years of follow-up (Figure 4). Nevertheless, pledgets might lead to a slight reduction of the EOA in the long run, but this finding requires external validation.

### Conflict of Interest Statement

Bart J. J. Velders: institutional research grant and speaker fees paid to his department by 10.13039/100004374Medtronic. Michiel D. Vriesendorp: institutional research grant and reimbursement of travel expenses from 10.13039/100004374Medtronic. Joseph F. Sabik III: North American Principal Investigator of the PERIGON Pivotal Trial for Medtronic. Francois Dagenais: speaker and consultant for Medtronic, COOK Medical, and Edwards Lifesciences. Louis Labrousse: research grant from Medtronic, 10.13039/100006520Edwards Lifesciences, and 10.13039/100000046Abbott. Vinayak Bapat: consultant for Medtronic, Edwards Lifesciences, and Abbott. Yaping Cai: employee of Medtronic. Robert J. M. Klautz: research support, consultation fees, and European Principal Investigator of the PERIGON Pivotal Trial for Medtronic. All other authors reported no conflicts of interest.

The *Journal* policy requires editors and reviewers to disclose conflicts of interest and to decline handling or reviewing manuscripts for which they may have a conflict of interest. The editors and reviewers of this article have no conflicts of interest.

## Figures and Tables

**Table E1:** IRB, IRB, and EC approval information—PERIGON Pivotal Trial

Site	IRB/REB/EC information	Date of IRB/REB/EC approval	IRB/REB/EC approval No.
United States			
Cleveland Clinic Cleveland, Ohio	Cleveland Clinic IRB 9500 Euclid Ave HSb 103 Cleveland, OH 44195	January 13, 2015	14-1537
Piedmont Hospital Atlanta, Georgia	Western IRB (WIRB) 1019 39th Ave SE Ste 120 Puyallup, WA 98374	September 10, 2014	20141211
University of Maryland Medical Center Baltimore, Maryland	Maryland School of Medicine IRB Human Research Protections Office 800 W Baltimore Street, Suite 100 Baltimore, MD 21201	April 30, 2015	HP-00063749
ProMedica Physicians Group Toledo, Ohio	Western IRB (WIRB) 1019 39th Ave SE Ste 120 Puyallup, WA 98374	August 28, 2014	20141211
Oklahoma Heart Hospital Oklahoma City, Oklahoma	Western IRB (WIRB) 1019 39th Ave SE Ste 120 Puyallup, WA 98374	October 17, 2014	20141211
Aurora Medical Group Cardiovascular and Thoracic Surgery Milwaukee, Wisconsin	Aurora Heath Care IRB Office 945 North 12th Street PO Box 342 W310 Milwaukee, WI 53201	August 19, 2014	14-77
Maimonides Medical Center Brooklyn, New York	Maimonides Medical Center IRB/Research Committee 4802 Tenth Ave Brooklyn, NY 11219	September 26, 2014	2014-08-17
University of Michigan Cardiovascular Center Ann Arbor, Michigan	University of Michigan, Office of Research University of Michigan Medical School 4107 Medical Science Building I 1301 Catherine Street SPC 5624 Ann Arbor, MI 48109-5624	September 11, 2014	IRB00001995
Cardiothoracic and Vascular Surgeons Austin, Texas	St David's Health Care IRB St David's Medical Center 919 East 32nd Street Austin, TX 78705	January 9, 2015	14-12-02
University of Colorado Aurora, Colorado	Colorado Multiple Institutional Review Board Campus Mailbox F490 13001 E 17th Place, Room N3214 Aurora, CO 80045	January 9, 2015	14-1348
University of Southern California Los Angeles, California	USC OPRS—Office for the Protection of Research Subjects General Hospital Suite 4700 1200 North State Street Los Angeles, CA 90033	September 15, 2014	HS-14-00527
University of Florida-Shands Gainesville, Florida	Western IRB 1019 39th Ave SE Ste 120 Puyallup, WA 98374	November 4, 2014	20141211
Houston Methodist Hospital Houston, Texas	Houston Methodist Institutional Review Board 6565 Fannin Street #MGJ6-014 Houston, TX 77030	September 9, 2014	0714-0157
University of Washington Seattle, Washington	Western IRB 1019 39th Ave SE Ste 120 Puyallup, WA 98374	November 30, 2014	20141211
Massachusetts General Hospital Boston, Massachusetts	Partners Human Research Committee 116 Huntington Avenue Ste 1002 Boston, MA 02116	January 28, 2015	2014P001477
Riverside Methodist Hospital Columbus, Ohio	Western IRB (WIRB) 1019 39th Ave SE Ste 120 Puyallup, WA 98374	August 21, 2014	20141211
Minneapolis Heart Institute Foundation Minneapolis, Minnesota	Quorum Review IRB 1501 Fourth Avenue Ste 800 Seattle, WA 98101	August 29, 2014	29584/1
New York Presbyterian Hospital/Columbia University Medical Center New York, New York	Columbia University IRB 154 Haven Ave, 1st Floor New York, NY 10032	May 22, 2015	IRB-AAAO9403
Mount Sinai Medical Center New York, New York	Program for the Protection of Human Subjects 345 E 102nd St Suite 200-2nd Floor New York, NY 10029	June 9, 2015	HS No: 15-00331
Stanford University Stanford, California	Research Compliance Office, Stanford University 3000 El Camino Real Five Palo Alto Square 4th Floor Palo Alto, CA 94306	November 17, 2015	4593
Hartford Hospital Hartford, Connecticut	Human Research Protection Program 80 Seymour Street PO Box 5037 Hartford, CT 06102-5037	December 3, 2020	HHC-2020-0335
Canada			
University of Ottawa Heart Institute Ottawa, Ontario, Canada	Ottawa Health Science Network Research Ethics Board (OHSN-REB) Ottawa Hospital, Civic Campus 725 Parkdale Avenue Civic Box 411 LOEB Building Ottawa, Ontario K1Y 4E9, Canada	August 18, 2014	20140100-01H
Toronto General Hospital Toronto, Ontario, Canada	UHN Research Ethics Board 700 University Ave Hyaro Building, Suite 1056 Toronto, Ontario M5G 1Z5, Canada	July 7, 2014	14-7354-A
Institut Universitaire de Cardiologie et de Pneumologie de Québec (IUCPQ) Quebec, Quebec, Canada	Comité d'ethique de la recherche IUCPQ Room U-4733, IRB 2725 chemin Ste-Foy Quebec G1V 4G5, Canada	June 30, 2014	2014-2354
Montreal Heart Institute Montreal, Quebec, Canada	Comité D’éthique de la Recherché Montreal Heart 5000 Rue Belanger est Montreal, Quebec H1T 1C8, Canada	July 17, 2014	2014-1686
London Health Sciences Centre London, Ontario, Canada	Western University Health Sciences Research Ethics Board 1393 Western Rd Support Services Building, Room 5182 London, Ontario N6G 1G9, Canada	June 7, 2016	107602
Europe			
Medizinische Hochschule Hannover Hannover, Germany	Central EC: Ethikkommission an der Technischen Universität München Ismaninger Straβe 22 81675 München, Germany Local EC: Ethikkommission der MHH Carl-Neuberg-Straβe 1 30625 Hannover, Germany	June 3, 2014	Reference: 36/14Mf-AS EUDAMED: CIV-14-01
Ospedale San Raffaele Milano, Italy	Comitato lini dell’ Ospedale San Raffaele Via Olgettina, 60 20132 Milano, Italy	March 6, 2014	Approval number not specified in approval letter
Hôpital Bichat—Claude Bernard Paris, France	Comité de protection des personnes Sud-Ouest et outre mer III Service de pharmacologie linique Groupe Hospitalier Pellegrin Bât 1A Place Amélie Raba Léon 33076 Bordeaux Cedex, France	January 29, 2014	ANSM number: 2013-A00897-38/4
Universitätsspital Zürich Zürich, Switzerland	Central EC: Kantonale Ethikkommission Bern (KEK) Institut für Pathophysiologie Hörsaaltrakt Pathologie, Eingang 43A, Büro H372 Murtenstrasse 31 3010 Bern, Switzerland Local EC: Kantonale Ethikkommission Zürich Stampfenbachstrasse 121 8090 Zürich, Switzerland	May 16, 2014	CEC number 010/14; SNCTP 17 CEC–ZH number: 2014–0068
Inselspital—Universitätsspital Bern Bern, Switzerland	Kantonale Ethikkommission Bern (KEK) Institut für Pathophysiologie Hörsaaltrakt Pathologie, Eingang 43A, Büro H372 Murtenstrasse 31 3010 Bern, Switzerland	May 16, 2014	CEC number: 010/14; SNCTP 17 CEC–ZH number: 2014–0068
Hôpital Haut-Lévêque—CHU de Bordeaux Bordeaux, France	Comité de protection des personnes Sud-Ouest et outre mer III Service de pharmacologie linique Groupe Hospitalier Pellegrin Bât. 1A Place Amélie Raba Léon 33076 Bordeaux Cedex, France	January 29, 2014	2013-A000897-38
Leids Universitair Medisch Centrum Leiden, The Netherlands	Medisch-Ethische Toetsingscommissie Leiden Den Haag Delft PO Box 9600 2300 RC Leiden, The Netherlands	March 21, 2014	P14.009/NL45419.058.13
Erasmus Medical Centre Rotterdam, The Netherlands	Medisch Ethische toetsings Commissie Erasmus MC Westzeedijk 353 Room Ae-337 3015 AA Rotterdam, The Netherlands	June 5, 2014	MEC-2014-272/NL45419.058.13
Universitätsklinikum Frankfurt Klinik für Thorax-, Herz- und Thorakale Gefäβchirurgie Frankfurt, Germany	Central EC: Ethikkommission der Fakultät für Medizin der Technischen Universität München Ismaninger Straβe 22 81675 München, Germany Local EC: Ethik- Kommission der Universitätsklinikum Frankfurt Theodor-Stern-Kai-7 60590 Frankfurt, Germany	June 3, 2014	Reference: 36/14Mf-AS EUDAMED: CIV-14-01
Guy's & St Thomas' NHS Foundation Trust–St Thomas' Hospital London, United Kingdom	NRES Committee London–Dulwich Health Research Authority Skipton House 80 London Road London SE1 6LH, United Kingdom	April 28, 2014	REC reference: 14/LO/0353 IRAS project ID: 134481
Universitätsklinikum Köln Köln, Germany	Central EC: Ethikkommission der Fakultät für Medizin der Technischen Universität München Ismaninger Straβe 22 81675 München, Germany Local EC: Ethikkommission der Medizinischen Fakultät der Universität zu Köln Kerpener Straβe 62 50937 Köln, Germany	June 3, 2014	Reference: 36/14Mf-AS EUDAMED: CIV-14-01
Herzzentrum Leipzig–Universitätsklinik Leipzig, Germany	Central EC: Ethikkommission der Fakultät für Medizin der Technischen Universität München Ismaninger Straβe 22 81675 München Germany Local EC: Ethikkommission an der Medizinischen Fakultät der Universität Leipzig Käthe-Kollwitz-Straβe 82 04109 Leipzig Germany	June 3, 2014	Reference: 36/14Mf-AS EUDAMED: CIV-14-01
Deutsches Herzzentrum München Klinik an der TU München München, Germany	Ethikkommission der Fakultät für Medizin der Technischen Universität München Ismaninger Straβe 22 81675 München, Germany	June 3, 2014	Reference: 36/14Mf-AS EUDAMED: CIV-14-01

Adapted from Klautz and colleagues,^7^ an Open Access article distributed under the terms of the Creative Commons Attribution-Noncommercial License. *IRB*, Institutional review board; *REB*, research ethics board; *EC*, ethics committee; *ANSM*, french national agency for medicines and health products safety; *CEC*, central ethics committee; *SNCTP*, swiss national clinical trials portal; *REC*, research ethics committee; *IRAS*, integrated research application system; *EUDAMED*, European database on medical devices.

**Table E2:** Clinical outcomes and hemodynamic performance at 30 days in the entire cohort

	Pledgets (n = 640)	Nonpledgets (n = 442)	*P* value∗
Composite endpoint (thromboembolism, endocarditis, and major PVL)	1.9% (1.1%-3.3%) (n = 12)	1.1% (0.5%-2.7%) (n = 5)	.34
Thromboembolism	1.4% (0.7%-2.7%) (n = 9)	1.1% (0.5%-2.7%) (n = 5)	.70
Endocarditis	0.3% (0.1%-1.2%) (n = 2)	0.0% (NA) (n = 0)	.24
Major PVL	0.2% (0.0%-1.1%) (n = 1)	0.0% (NA) (n = 0)	.41
All PVL	0.2% (0.0%-1.1%) (n = 1)	0.2% (0.0%-1.6%) (n = 1)	.79
Major hemorrhage	1.1% (0.5%-2.3%) (n = 7)	0.9% (0.3%-2.4%) (n = 4)	.76
All-cause mortality	0.8% (0.3%-1.9%) (n = 5)	1.1% (0.5%-2.7%) (n = 5)	.55
Cardiac-related mortality	0.6% (0.2%-1.7%) (n = 4)	0.5% (0.1%-1.8%) (n = 2)	.71
Valve-related mortality	0.0% (NA) (n = 0)	0.0% (NA) (n = 0)	NA
Reintervention	0.6% (0.2%-1.7%) (n = 4)	0.0% (NA) (n = 0)	.10
Explant	0.6% (0.2%-1.7%) (n = 4)	0.0% (NA) (n = 0)	.10
Permanent pacemaker implantation	3.3% (2.2%-5.0%) (n = 21)	4.8% (3.1%-7.2%) (n = 21)	.22
Mean pressure gradient, mm Hg	12.9 ± 4.4	13.4 ± 5.0	.14
Peak pressure gradient, mm Hg	23.7 ± 7.9	24.3 ± 8.8	.25
EOA, cm^2^	1.60 ± 0.38	1.58 ± 0.38	.46
EOAi, cm^2^/m^2^	0.80 ± 0.19	0.81 ± 0.20	.79
Prosthesis-patient mismatch, n (%)			.36
None	269 (49.9)	170 (45.1)	
Moderate	193 (35.8)	148 (39.3)	
Severe	77 (14.3)	59 (15.6)	

Clinical outcomes are reported as 5-year Kaplan–Meier event rates including 95% CI. Hemodynamic performance is presented either as mean ± SD or median (interquartile range). *PVL*, Paravalvular leak; *NA*, not applicable; *EOA*, effective orifice area; *EOAi*, effective orifice area indexed according to body surface area.

∗ *P* value from log rank test for all clinical outcomes and from an independent samples *t* test or Mann–Whitney *U* test for echocardiographic data.

**Table E3:** Clinical outcomes and hemodynamic performance at 5 years of follow-up in the entire cohort

	Pledgets (n = 640)	Nonpledgets (n = 442)	*P* value∗
Composite endpoint (thromboembolism, endocarditis, and major PVL)	9.2% (7.1%-12.0%) (n = 53)	10.2% (7.6%-13.6%) (n = 41)	.59
Thromboembolism	4.5% (3.1%-6.4%) (n = 27)	6.9% (4.8%-10.0%) (n = 27)	.17
Endocarditis	5.0% (3.4%-7.3%) (n = 26)	3.8% (2.3%-6.2%) (n = 15)	.55
Major PVL	0.3% (0.1%-1.3%) (n = 2)	0.0% (NA) (n = 0)	.24
All PVL	1.0% (0.4%-2.2%) (n = 6)	1.3% (0.5%-3.6%) (n = 4)	.92
All-cause mortality	12.0% (9.5%-15.1%) (n = 67)	12.0% (9.1%-15.6%) (n = 48)	.93
Cardiac-related mortality	5.8% (4.1%-8.3%) (n = 31)	5.7% (3.8%-8.6%) (n = 22)	.98
Valve-related mortality	1.7% (0.9%-3.2%) (n = 10)	1.0% (0.4%-2.6%) (n = 4)	.34
Reintervention	2.7% (1.7%-4.5%) (n = 16)	3.5% (2.0%-6.0%) (n = 13)	.70
Explant	2.6% (1.6%-4.3%) (n = 15)	2.9% (1.6%-5.2%) (n = 11)	.91
Permanent pacemaker implantation	6.9% (5.2%-9.3%) (n = 42)	7.5% (5.3%-10.6%) (n = 31)	.76
Mean pressure gradient, mm Hg	12.7 ± 4.9	12.3 ± 4.1	.48
Peak pressure gradient, mm Hg	22.5 ± 8.3	22.0 ± 7.6	.54
EOA, cm^2^	1.40 ± 0.33	1.45 ± 0.36	.19
EOAi, cm^2^/m^2^	0.71 ± 0.16	0.75 ± 0.18	.06
Prosthesis-patient mismatch, n (%)			.21
None	64 (33.3)	49 (32.2)	
Moderate	70 (36.5)	68 (44.7)	
Severe	58 (30.2)	35 (23.0)	

Clinical outcomes are reported as 5-year Kaplan–Meier event rates including 95% CI. Hemodynamic performance is presented either as mean ± SD or median (interquartile range). *PVL*, Paravalvular leak; *NA*, not applicable; *EOA*, effective orifice area; *EOAi*, effective orifice area indexed according to body surface area.

∗ *P* value from log rank test for all clinical outcomes and from an independent samples *t* test, Mann–Whitney *U* test, or χ^2^ test for echocardiographic data.

**Table E4:** Clinical outcomes and hemodynamic performance at 30 days in the propensity score-matched cohort

	Pledgets (n = 397)	Nonpledgets (n = 397)	*P* value∗
Composite endpoint (thromboembolism, endocarditis, and major PVL)	2.8% (1.5%-5.0%) (n = 11)	1.0% (0.4%-2.7%) (n = 4)	.07
Thromboembolism	2.0% (1.0%-4.0%) (n = 8)	1.0% (0.4%-2.7%) (n = 4)	.25
Endocarditis	0.5% (0.1%-2.0%) (n = 2)	0.0% (NA) (n = 0)	.16
Major PVL	0.3% (0.0%-1.8%) (n = 1)	0.0% (NA) (n = 0)	.34
All PVL	0.3% (0.0%-1.8%) (n = 1)	0.3% (0.0%-1.8%) (n = 1)	>.99
Major hemorrhage	0.8% (0.2%-2.3%) (n = 3)	1.0% (0.4%-2.7%) (n = 4)	.71
All-cause mortality	1.0% (0.4%-2.7%) (n = 4)	1.0% (0.4%-2.7%) (n = 4)	.99
Cardiac-related mortality	1.0% (0.4%-2.7%) (n = 4)	0.3% (0.0%-1.8%) (n = 1)	.18
Valve-related mortality	0.0% (NA) (n = 0)	0.0% (NA) (n = 0)	NA
Reintervention	0.8% (0.2%-2.3%) (n = 3)	0.0% (NA) (n = 0)	.08
Explant	0.8% (0.2%-2.3%) (n = 3)	0.0% (NA) (n = 0)	.08
Permanent pacemaker implantation	2.3% (1.2%-4.3%) (n = 9)	4.3% (2.7%-6.8%) (n = 17)	.11
Mean pressure gradient, mm Hg	12.7 ± 4.4	13.5 ± 5.1	.010
Peak pressure gradient, mm Hg	23.3 ± 7.9	24.6 ± 9.0	.027
EOA, cm^2^	1.55 (0.80-2.84)	1.54 (0.70-3.01)	.99
EOAi, cm^2^/m^2^	0.79 (0.38-1.41)	0.79 (0.31-1.50)	.88
Prosthesis-patient mismatch, n (%)			.87
None	158 (47.2)	155 (45.2)	
Moderate	127 (37.9)	134 (39.1)	
Severe	50 (14.9)	54 (15.7)	

Clinical outcomes are reported as 5-year Kaplan–Meier event rates including 95% CI. Hemodynamic performance is presented either as mean ± SD or median (interquartile range). *PVL*, Paravalvular leak; *NA*, not available; *EOA*, effective orifice area; *EOAi*, effective orifice area indexed according to body surface area.

∗ *P* value from log rank test for all clinical outcomes and from an independent samples *t* test, Mann–Whitney *U* test, or χ^2^ test for echocardiographic data.

**Table E5:** Baseline and procedural characteristics in valve sizes <23 mm

	Pledgets (n = 131)	Nonpledgets (n = 112)	*P* value
Age, y	70.9 ± 7.1	73.4 ± 10.3	.035
Male sex	51 (38.9)	40 (35.7)	.61
Body surface area, m^2^	1.8 ± 0.2	1.8 ± 0.2	.19
Body mass index	29.3 ± 5.9	28.8 ± 6.6	.49
NYHA classification III-IV	63 (48.1)	54 (48.2)	.98
STS risk of mortality, %	2.1 ± 1.3	2.8 ± 1.9	.002
Diabetes	42 (32.1)	26 (23.2)	.13
Hypertension	99 (75.6)	84 (75.0)	.92
Peripheral vascular disease	11 (8.4)	7 (6.3)	.52
Renal dysfunction/insufficiency	12 (9.2)	17 (15.2)	.15
Stroke/CVA	11 (8.4)	5 (4.5)	.22
COPD	9 (6.9)	13 (11.6)	.20
Left ventricular ejection fraction, %	62.7 ± 7.2	61.6 ± 7.1	.35
Coronary artery disease	59 (45.0)	44 (39.3)	.37
Left ventricular hypertrophy	55 (42.0)	34 (30.4)	.06
Atrial fibrillation	10 (7.6)	14 (12.5)	.21
Isolated/mixed aortic stenosis	126 (96.2)	111 (99.1)	.22
Minimally invasive surgical approach	36 (27.9)	22 (20.0)	.16
Concomitant procedures			
None	64 (48.9)	73 (65.2)	.011
CABG	45 (34.4)	28 (25.0)	.11
Ascending aortic aneurysm not requiring circulatory arrest	5 (3.8)	0 (.0)	.06
Other∗	32 (24.4)	18 (16.1)	.11
Annular calcification	111 (84.7)	95 (84.8)	.98
Total bypass time, min	102.8 ± 37.5	93.1 ± 39.2	.05
Aortic crossclamp time, min	78.6 ± 29.4	69.2 ± 31.3	.017
Valve size implanted			.042
17 mm	0 (0.0)	1 (.9)	
19 mm	16 (12.2)	23 (2.5)	
21 mm	115 (87.8)	88 (78.6)	
Mean pressure gradient, mm Hg	42.9 ± 16.9	46.5 ± 17.3	.11
Effective orifice area, cm^2^	1.17 (0.65-2.14)	1.17 (0.68-1.73)	.86
Indexed effective orifice area, cm^2^/m^2^	0.38 (0.19-1.19)	0.39 (0.20-1.22)	.74

Data are presented as either mean ± SD, median (interquartile range), or n (%) and compared with the independent samples *t* test, Mann–Whitney *U* test, or χ^2^/Fisher exact test, respectively. *NYHA*, New York Heart Association; *STS*, Society of Thoracic Surgeons; *CVA*, cerebrovascular accident; *COPD*, chronic obstructive pulmonary disease; *CABG*, coronary artery bypass grafting.

∗ Includes implantable cardiac device, left atrial appendage closure, patent foramen ovale closure, resection of subaortic membrane not requiring myectomy, and dissection repair not requiring circulatory arrest.

**Table E6:** Baseline and procedural characteristics within the nonpledgeted subgroups

	Mattress∗ (n = 180)	Nonmattress† (n = 205)	*P* value
Age, y	71.0 ± 8.6	72.3 ± 8.9	.15
Male sex	134 (74.4)	149 (72.7)	.70
Body surface area, m^2^	2.0 ± 0.2	1.9 ± 0.2	.14
Body mass index	29.2 ± 5.3	28.2 ± 5.1	.046
NYHA classification III-IV	96 (53.3)	82 (40.0)	.009
STS risk of mortality, %	2.2 ± 1.5	2.3 ± 1.7	.50
Diabetes	56 (31.1)	43 (21.0)	.023
Hypertension	140 (77.8)	134 (65.4)	.007
Peripheral vascular disease	18 (10.0)	17 (8.3)	.56
Renal dysfunction/insufficiency	26 (14.4)	12 (5.9)	.005
Stroke/CVA	12 (6.7)	4 (2.0)	.037
COPD	13 (7.2)	30 (14.6)	.021
Left ventricular ejection fraction, %	59.9 ± 8.4	57.7 ± 11.5	.06
Coronary artery disease	91 (50.6)	70 (34.1)	.001
Left ventricular hypertrophy	56 (31.1)	91 (44.4)	.008
Atrial fibrillation	29 (16.1)	24 (11.7)	.21
Isolated/mixed aortic stenosis	175 (97.2)	199 (97.1)	.93
Minimally invasive surgical approach	23 (12.9)	27 (13.2)	.93
Concomitant procedures			
None	83 (46.1)	133 (64.9)	<.001
CABG	60 (33.3)	59 (28.8)	.33
Ascending aortic aneurysm not requiring circulatory arrest	16 (8.9)	5 (2.4)	.005
Other‡	41 (22.8)	14 (6.8)	<.001
Annular calcification	153 (85.0)	167 (81.5)	.36
Total bypass time, min	103.3 ± 42.4	103.2 ± 37.7	.97
Aortic crossclamp time, min	79.4 ± 34.6	77.2 ± 30.7	.51
Valve size implanted			.40
17 mm	1 (0.6)	0 (0.0)	
19 mm	6 (3.3)	15 (7.3)	
21 mm	41 (22.8)	39 (19.0)	
23 mm	64 (35.6)	82 (4.0)	
25 mm	53 (29.4)	55 (26.8)	
27 mm	13 (7.2)	13 (6.3)	
29 mm	2 (1.1)	1 (0.5)	
Mean pressure gradient, mm Hg	43.4 ± 16.8	45.2 ± 16.6	.30
Effective orifice area, cm^2^	0.78 (0.35-2.79)	0.73 (0.38-3.43)	.41
Indexed effective orifice area, cm^2^/m^2^	0.39 (0.20-1.65)	0.38 (0.18-1.82)	.48

Data are presented as either mean ± standard deviation, median (interquartile range), or n (%) and compared with the independent samples *t* test, Mann–Whitney *U* test, or χ^2^/Fisher exact test, respectively, except where otherwise noted. *NYHA*, New York Heart Association; *STS*, Society of Thoracic Surgeons; *CVA*, cerebrovascular accident; *COPD*, chronic obstructive pulmonary disease; *CABG*, coronary artery bypass grafting.

∗ The mattress group consisted of everting and noneverting mattress sutures.

† The nonmattress group comprised simple interrupted and continuous sutures.

‡ Includes implantable cardiac device, left atrial appendage closure, patent foramen ovale closure, resection of subaortic membrane not requiring myectomy, and dissection repair not requiring circulatory arrest.

**Table E7:** Hemodynamic performance at discharge up to 30 days and at 5 years of follow-up within the nonpledgeted subgroups

	Mattress∗ (n = 180)	Nonmattress† (n = 205)	*P* value
Mean pressure gradient, mm Hg			
Discharge up to 30 days	13.2 ± 5.1	13.9 ± 5.0	.18
5 years	12.5 ± 4.3	12.6 ± 4.1	.84
Peak pressure gradient, mm Hg			
Discharge up to 30 days	23.8 ± 8.7	25.0 ± 9.1	.20
5 years	22.4 ± 7.2	22.5 ± 8.2	.90
Effective orifice area, cm^2^			
Discharge up to 30 days	1.60 (0.70-3.01)	1.51 (0.80-2.64)	.16
5 years	1.44 (0.86-2.44)	1.38 (0.79-2.44)	.20
Indexed effective orifice area, cm^2^/m^2^			
Discharge up to 30 days	0.79 (0.31-1.50)	0.78 (0.41-1.62)	.44
5 years	0.78 (0.41-1.31)	0.72 (0.45-1.18)	.25
Prosthesis-patient mismatch			
Discharge up to 30 days			.85
None	72 (46.8)	77 (44.0)	
Moderate	58 (37.7)	71 (4.6)	
Severe	24/154 (15.6)	27/175 (15.4)	
5 years			.60
None	22 (36.1)	20 (28.2)	
Moderate	27 (44.3)	34 (47.9)	
Severe	12 (19.7)	17 (23.9)	
Paravalvular leak			
Discharge up to 30 days			.46
None	125 (73.5)	154 (77.8)	
Trace	30 (17.6)	32 (16.2)	
Mild	15 (8.8)	11 (5.6)	
Moderate	0 (0.0)	1 (.5)	
Severe	0 (0.0)	0 (.0)	
5 years			.22
None	60 (88.2)	70 (85.4)	
Trace	3 (4.4)	9 (11.0)	
Mild	5 (7.4)	3 (3.7)	
Moderate	0 (0.0)	0 (0.0)	
Severe	0 (0.0)	0 (0.0)	

Numerical data are presented as mean ± SD or median (interquartile range) according to their distribution, and categorical data are summarized as n (%); data were compared using the independent samples *t* test, Mann–Whitney *U* test, and χ^2^ test/Fisher exact test, respectively.

∗ The mattress group consisted of everting and noneverting mattress sutures.

† The nonmattress group comprised simple interrupted and continuous sutures.
